# The Suppression of Fraudulent Diplomas

**Published:** 1880-06

**Authors:** 


### EDITORIAL, ETC.

*Suppression of Fraudulent Diplomas.*—For a number of years past Philadelphia has been the seat of several bogus Colleges which have been flooding this and foreign countries with fraudulent medical and dental diplomas. The Legislature of Pennsylvania tried in 1872 to suppress the institutions, but failed, and they have been run ever since on legally issued charters. The authorities declared themselves powerless to stop it. Six months ago the city editor of the *Record*, John Morris, called the attention of Attorney-General Palmer to these concerns and the grave necessity for their suppression. But the State had no evidence against them and the Attorney-General had no money with which to proceed. Then the *Record* offered to advance the funds to the Commonwealth upon promise of reimbursement by the State Legislature, and the work commenced. The other day it culminated in proceedings which will suppress five bogus medical colleges, and in the arrest of John Buchanan, dean of the American University, of Philadelphia, and the Eclectic Medical College of Pennsylvania. He was also President of the National Eclectic Medical Association, which issued diplomas, and, under the alias of James Murray, D. D., he acted as dean of a concern issuing its diplomas as the Livingston University of America. Two others of the faculty, Charles S. Polk and John J. Seggins, were arrested, while six others of the faculty are still at large, apparently out of the reach of the police.

The City Editor of the *Record* has prepared evidence which it is alleged will show the sales of forty-two diplomas to various persons. He gives the names of eleven others to whom the diplomas were offered, and the names of eleven agents who were acting for Buchanan. The *Record* man himself set the trap which secured Buchanan. For $75 he obtained three medical diplomas, one in English, from the Eclectic Medical College of Pennsylvania, a regularly chartered institution ; one from the American University of Philadelphia, in English, and another from the National Eclectic Medical Association.

One feature of the proceeding is that while the last-named concern was organized in 1879, its diploma to the newspaper man is dated 1878. All purported to show that the newspaper man, under the name of Dr. John Fanning, of Tippecanoe City, Ohio, studied medicine for three years, had attended two full courses of lectures, and had passed a satisfactory examination in each of the seven branches of medicine. Not satisfied with this evidence, he sent letters under the name of Dr. George A. Dawson, apparently belonging to Chester Court-House, S. C., asking for degrees. For $150 he obtained five degrees, two of doctor of medicine, one doctor of divinity, one doctor of law, and one doctor of civil law.

The final letter in this transaction passed through the mail, Dr. Buchanan receipting for it. He also deposited the diplomas in the mail to be delivered to the spurious doctor in South Carolina. Special Postal Agent Barrett, Chief of Police Given, a Deputy United States Marshal, and the newspaper man walked into Buchanan's place this afternoon, arrested him, and then captured about half a ton of bogus diplomas, with a mass of correspondence, showing the traffic in diplomas and the sale of about 3,000 sheepskins.

These bogus College faculties have been selling diplomas "for a consideration" to every part of the world, but especially to Germany. John Buchanan, the alleged chief offender, was committed by the United States Commissioner in default of $10,000 bail on a charge of using the mails in connection with the business. The final exposure and arrest were due, as before stated, to the enterprise and public spirit of Mr. John Morris, City Editor of the Philadelphia *Record*, who accomplished what the legal authorities have repeatedly failed to achieve. Recently the matter has been brought conspicuously before the public by some correspondence of President A. D. White, of Cornell University, at present United States minister to Germany and which was published in a recent number of this Journal. Mr. White resented some allusions in a popular farce to quacks created by American ready-made diplomas, but at the same time admitted and deprecated this traffic in spurious degrees. The matter was, however, exposed and ventilated several years ago by the New York *Nation*. On that occasion one of the persons accused in the present case proved that he represented a regularly chartered college, with authority to grant diplomas, and threatened a suit for libel. In the course of the controversy which ensued it came out that Germany's skirts were not clean in regard to these same practices about which its people bring such loud accusations against this Country. It was proved that the great University of Leipsic had been in the habit of vending its diplomas to liberal bidders, and three cases were cited of such diplomas granted within the year. The practice, however, has been entirely broken up in Germany, and it is to be hoped that the present exposures and prosecutions will end in breaking it up in this country also. The evils caused by this system are very numerous and very great. The fraudulent character of the diploma, heinous as that is in a moral point of view, is the least of these evils. The system is injurious in the highest degree to regular medical and dental colleges, which conscientiously aim to give thorough and scientific instruction. It is discouraging to all regular practitioners, who have prepared themselves for the business of their life by long and laborious studies. When a man has studied medicine or dentistry for years, it is simply disheartening to him to find a pretender, who may scarcely have read anything, put on an equality with him by a spurious diploma.

We are glad to learn that there is every reason to believe that this sale of diplomas has been suppressed and the offenders brought to justice.

